# Diminished ovarian reserve in systemic lupus erythematosus: clinical, immunological, viral, and therapeutic determinants in a 24-month prospective study

**DOI:** 10.3389/fimmu.2026.1918504

**Published:** 2026-10-14

**Authors:** Margherita Rizzo, Silvia Beltrami, Francesca Ruffilli, Carlo Garaffoni, Ettore Silvagni, Daria Bortolotti, Giorgia Cianci, Maila Galloppa, Beatrice Maranini, Marcello Govoni, Roberta Rizzo, Alessandra Bortoluzzi

**Affiliations:** 1 Rheumatology Unit, Azienda Ospedaliero-Universitaria Sant’Anna, Cona, Ferrara, Italy; 2 Department of Medical Sciences, University of Ferrara, Ferrara, Italy; 3 Department of Environmental and Prevention Sciences, University of Ferrara, Ferrara, Italy

**Keywords:** anti-Müllerian hormone, belimumab, diminished ovarian reserve, hydroxychloroquine, reproductive health, systemic lupus erythematosus

## Abstract

**Background and objectives:**

Systemic lupus erythematosus (SLE) predominantly affects women of reproductive age; however, the impact of the disease, chronic inflammation, and its treatments on ovarian reserve remains insufficiently characterized. This study aimed to assess the prevalence of diminished ovarian reserve in SLE and to evaluate the longitudinal associations of clinical characteristics, therapeutic exposures, and herpesvirus serologies with Anti-Müllerian Hormone (AMH) levels.

**Methods:**

This observational prospective monocentric study followed 80 premenopausal women (18–45 years) with SLE for 24 months. Clinical data, treatments, AMH levels, and herpesvirus serologies were collected every 6 months. Patients were stratified by diminished ovarian reserve (AMH <1 ng/mL). Longitudinal AMH trajectories and the impact of covariates were evaluated using repeated-measures analyses and linear mixed-effects models.

**Results:**

The cohort exhibited a high prevalence of diminished ovarian reserve (45% of the study population), associated with older age (37 ± 7 vs. 34 ± 7 years, p = 0.041), longer disease duration (14 ± 9 vs. 10 ± 6 years, p = 0.026), higher cumulative damage (p = 0.007), and lupus anticoagulant positivity (14% vs. 39%, p=0.009). Disease activity was comparable across groups. Hydroxychloroquine use was more frequent in women without diminished ovarian reserve (89% vs 69%, p = 0.033). Over 24 months, AMH levels remained stable with no progressive decline. Belimumab exposure did not adversely affect AMH trajectories, and viral serologies showed no direct long-term impact on AMH levels.

**Conclusions:**

This prospective longitudinal study highlights that diminished ovarian reserve is common in women with SLE. AMH levels remained stable over 24 months, with no adverse longitudinal association with belimumab exposure. Hydroxychloroquine use was associated with a lower prevalence of diminished ovarian reserve and warrants further investigation. Serial AMH assessment may support personalized reproductive counselling in women with SLE.

## 1 Introduction

Infertility, diminished ovarian reserve and premature ovarian insufficiency are interrelated medical conditions affecting women’s reproductive health significantly. Infertility is clinically defined as the inability to achieve conception after one year of regular, unprotected intercourses during the reproductive age (1). Although there is no universally accepted definition of diminished ovarian reserve, it generally refers to ovarian reserve lower than expected for age, typically in the presence of regular menstrual cycles. Diminished ovarian reserve can be assessed using ovarian reserve markers, including serum anti-Müllerian hormone (AMH), follicle-stimulating hormone (FSH), and antral follicle count (AFC), although thresholds for abnormal ovarian reserve are not universally established (2). Premature ovarian insufficiency (POI), instead, is defined by the occurrence of a combination of a 4-to-6-month period of amenorrhea and measurement of elevated FSH in women < 40 years not using contraceptives (3). Hormonal biomarkers such as AMH, a glycoprotein hormone primarily produced by granulosa cells in developing ovarian follicles, and FSH are routinely employed to evaluate ovarian reserve. Clinically, AMH levels are useful in ascertaining ovarian reserve because they correlate well with the number of remaining follicles (4).

Among infectious contributors to reproductive dysfunction, herpesviruses have been implicated in infertility and adverse pregnancy outcomes through mechanisms that include chronic inflammation, hormonal dysregulation, and direct effects on endometrial and trophoblastic tissues. Persistent inflammation, in particular, disrupts the immune and hormonal balance necessary for ovulation, embryo implantation, and pregnancy maintenance (5, 6). Human herpesvirus 6 (HHV-6) has been shown to alter the local immune environment and promote the overactivation of natural killer (NK) cells, factors linked to implantation failure. In addition it could infect endometrial epithelial cells and disrupt local immune tolerance, contributing to hormonal alterations and menstrual disorders that compromise fertility (7). Epstein-Barr virus (EBV), similarly, may contribute to infertility by inducing immune dysregulation and promoting NK cell overactivity, creating a hostile uterine environment and raising the risk of implantation failure and early pregnancy loss (8). CMV can impair trophoblast invasion and disrupt spiral artery remodeling, both essential for placental development, thereby increasing the risk of pregnancy complications (9). Finally, although less extensively studied, HHV-7 and HHV-8 may also impair fertility through systemic inflammation and ovarian dysfunction (7).

SLE has a significantly higher prevalence in women, particularly during their reproductive years. As disease onset often overlaps with peak fertility age, understanding the impact of SLE on reproductive health is essential. Although data remain limited, growing evidence indicates that fertility in women with SLE may be compromised by a complex interplay of intrinsic factors such as disease activity, lupus flares, cumulative organ damage and psychosocial and physical consequences of the disease and extrinsic factors, including the gonadotoxic effects of some immunosuppressive therapies (10). Regarding the role of immunosuppressive therapy, multiple studies have established that exposure to cyclophosphamide (CYC) is a major risk factor for sustained amenorrhea and the subsequent development of POI (10). The gonadotoxicity of CYC is dose-dependent and becomes particularly significant in young women, complicating the interplay between disease control and preservation of fertility. In response to this challenge, adjunctive treatment strategies using gonadotropin-releasing hormone (GnRH) analogues have been employed. When administered concurrently with CYC, GnRH analogues create a hypoestrogenic state that appears to protect ovarian reserve and reduce the incidence of POI (11). Taken together, these mechanisms can impair gonadal function and reduce overall reproductive potential, which may result in infertility (10). Consistent with this negative impact on ovarian reserve, several studies have established a correlation between SLE and reduced AMH levels, reporting that premenopausal women with SLE exhibit lower serum AMH levels compared to healthy controls, suggesting a negative impact on ovarian reserve (12, 13).

The present prospective study was designed to comprehensively assess diminished ovarian reserve in women of reproductive age with SLE by integrating clinical, hormonal, and viral serological data. We also evaluated whether clinical characteristics, treatments, and herpetic serological profiles were associated with reduced ovarian function over a 24-month follow-up. With this integrated approach, we aimed to better delineate the immune–infectious and therapeutic determinants of reproductive dysfunction in women with SLE.

## 2 Methods

### 2.1 Study design and population

This was a prospective, observational, single-center study conducted at the Lupus Clinic of the University Hospital of Ferrara between 2019 and 2023. The study population consisted of women of childbearing age (18–45 years) with a confirmed diagnosis of SLE, established according to the 2012 SLICC, and/or 2019 EULAR/ACR classification criteria (14, 15). Inclusion criteria required participants to be aged 18–45 years and to provide written informed consent. All patients were receiving immunomodulating (hydroxychloroquine, HCQ) or immunosuppressive treatment, excluding cyclophosphamide (CYC). Patients assigned to belimumab treatment complied with prescription rules defined by the Emilia-Romagna district, requiring ANA-positive SLE with active disease (SELENA-SLEDAI score≥10) or positive anti-dsDNA antibodies, low C3 or C4, and inadequate response to standard-of-care (residual disease activity for ≥3 months preventing glucocorticoid tapering to ≤7.5mg/day) or frequent relapses.

### 2.2 Assessments and outcome

At the inclusion visit, demographics, ethnicity, body mass index (BMI), date of SLE diagnosis, clinical and serological characteristics, comorbidities, SLICC/ACR Damage Index (SDI) (16), and previous treatments were recorded. Obstetric and reproductive history were assessed, including the number of full-term pregnancies (both uncomplicated and complicated) and a history of amenorrhea lasting ≥6 months. At each study visit (baseline and at 6, 12, 18, and 24 months), we recorded disease activity using the SLE Disease Activity Index 2000 (SLEDAI-2K) and the Physician Global Assessment (PGA), along with serology for anti-dsDNA antibodies, C3, C4 and concomitant treatments (17, 18). At the same time points, AMH levels and viral serology were collected, including IgG and IgM antibodies against Epstein–Barr virus (EBV), cytomegalovirus (CMV), human herpesvirus 6 (HHV-6), HHV-7, and HHV-8. AMH Serum concentrations were quantified using commercially available enzyme-linked immunosorbent assay (ELISA) kits according to the manufacturers’ instructions. AMH levels were measured using kits from MyBioSource (San Diego, CA, USA; Cat. No. MBS262919). Viral serologies (IgG and IgM for HHV-6, HHV-7, HHV-8, EBV [EBNA-1], and CMV) were assessed using ELISA kits from the same manufacturer (MyBioSource, San Diego, CA, USA; corresponding catalogue numbers: MBS2800429, MBS2610945, MBS1610833, MBS1610832, MBS167810, MBS3803087, MBS495647, MBS495646, MBS580099, MBS495663).

### 2.3 Ethics approval

The study was approved by the Ethics Committee of the Emilia Romagna Region, Central Emilia Area (Institutional Review Board code 842/2019/Oss/AOUFe) and was conducted in accordance with the Declaration of Helsinki. All data were recorded and managed using a secure electronic database (REDCap).

### 2.4 Statistical analysis

Continuous variables were summarized as mean (standard deviation [SD]) or median (interquartile range [IQR]), as appropriate, and categorical variables as counts and percentages. A stratified analysis was performed according to the presence of diminished ovarian reserve, defined as AMH levels <1 ng/mL (4, 19–21). Demographic, clinical, and serological variables were evaluated for their association with diminished ovarian reserve. Between-group differences were assessed using the Welch’s t-test or Mann–Whitney U test for continuous variables, and the chi-square or Fisher’s exact test for categorical variables, as appropriate.

Exploratory longitudinal analyses were conducted using repeated-measures ANOVA (with Greenhouse–Geisser correction when sphericity was violated) or the Friedman test, as appropriate; Cochran’s Q test was used for binary outcomes. To identify factors associated with longitudinal changes in AMH levels, multivariable linear mixed-effects models were constructed, including time as an independent variable, relevant clinical covariates (e.g., baseline age, disease activity, and treatments), and interaction terms between time and covariates. All interactions were initially tested, and only those statistically significant were retained in the final models. Additional models were fitted to evaluate the impact of viral serology (HHV-6, HHV-7, HHV-8, CMV, EBV) on AMH trajectories.

All tests were two-sided, with p<0.05 considered statistically significant. R version 4.5.1 (R Foundation for Statistical Computing) was used for analyses.

## 3 Results

Baseline characteristics of the study population (n=80), stratified by diminished ovarian reserve status, are presented in **Table 1**. The mean age was 35 ± 7 years, and the mean body mass index (BMI) was 23.8 ± 5.5. Disease duration averaged 12 ± 8 years. Most participants were Caucasian (75, 94%), and 15 patients (19%) reported at least one comorbidity, most commonly endocrine-metabolic disorders (26, 32%), chronic gastrointestinal-liver-biliary diseases (23, 28%), and cardiovascular diseases (19, 23%). Regarding reproductive history, the majority of patients had no prior pregnancies (56%). Most patients reported no history of amenorrhea (71%), and only 4 patients (5%) experienced amenorrhea lasting ≥6 months.

**Table 1:** Baseline characteristics of the study population divided by diminished ovarian reserve.

Characteristic	Overall N = 80*^1^*	Diminished ovarian reserve		Effect size (95% CI)	P-value*^2^*
		No N = 44*^1^*	Yes N = 36*^1^*		
Age	35 (7)	34 (7)	37 (7)	SMD (d) = -0.5 (-0.9, 0)	**0.041**
Ethnicity				V= 0 (0, 1)	>0.9
*Arabic*	2 (2.5%)	1 (2.3%)	1 (2.8%)		
*Black/African-American*	2 (2.5%)	1 (2.3%)	1 (2.8%)		
*Caucasian*	75 (94%)	41 (93%)	34 (94%)		
*Latin-American*	1 (1.3%)	1 (2.3%)	0 (0%)		
BMI	23.8 (5.5)	23.4 (5.8)	24.3 (5.1)	SMD (d) = -0.2 (-0.6, 0.3)	0.4
Disease duration (years)	12 (8)	10 (6)	14 (9)	SMD (d) = -0.5 (-1, -0.1)	**0.026**
Any comorbidity	15 (19%)	11 (25%)	4 (11%)	OR= 2.7 (0.8, 9.2)	0.11
Cardiovascular diseases	19 (24%)	9 (20%)	10 (28%)	OR= 0.7 (0.2, 1.9)	0.4
Cerebrovascular diseases	1 (1.3%)	1 (2.3%)	0 (0%)	-	>0.9
Nervous system/psychiatric diseases	15 (19%)	9 (20%)	6 (17%)	OR= 1.3 (0.4, 4)	0.7
Chronic skin diseases	6 (7.5%)	3 (6.8%)	3 (8.3%)	OR= 0.8 (0.2, 4.3)	>0.9
Chronic Pulmonary-upper respiratory diseases	6 (7.5%)	3 (6.8%)	3 (8.3%)	OR= 0.8 (0.2, 4.3)	>0.9
Chronic Gastrointestinal-liver-biliary diseases	23 (29%)	11 (25%)	12 (33%)	OR= 0.7 (0.3, 1.8)	0.4
Endocrinology-metabolic diseases	25 (31%)	17 (39%)	8 (22%)	OR= 2.2 (0.8, 5.9)	0.12
Kidney-Urological diseases	3 (3.8%)	1 (2.3%)	2 (5.6%)	OR= 0.4 (0, 4.5)	0.6
Hematological diseases	10 (13%)	4 (9.1%)	6 (17%)	OR= 0.5 (0.1, 1.9)	0.3
Eye-disorder related diseases	6 (7.5%)	2 (4.5%)	4 (11%)	OR= 0.4 (0.1, 2.2)	0.4
Infectious diseases	3 (3.8%)	1 (2.3%)	2 (5.6%)	OR= 0.4 (0, 4.5)	0.6
Gynecological diseases	13 (16%)	4 (9.1%)	9 (25%)	OR= 0.3 (0.1, 1.1)	0.055
Musculoskeletal diseases	13 (16%)	5 (11%)	8 (22%)	OR= 0.4 (0.1, 1.5	0.2
Obstetric history				V= 0 (0, 1)	0.4
*No pregnancy*	45 (56%)	27 (61%)	18 (50%)		
*Complicated pregnancies*	17 (21%)	7 (16%)	10 (28%)		
*Uncomplicated pregnancies*	18 (23%)	10 (23%)	8 (22%)	OR= 0.9 (0.3, 2.3)	
No history of amenorrhea	57 (71%)	32 (73%)	25 (69%)	OR= 0.9 (0.3, 2.3)	0.7
History of amenorrhea ≥ 6 months	4 (5.0%)	1 (2.3%)	3 (8.3%)	OR= 3.9 (0.4, 39.3)	0.3

^1^ Mean (SD); n (%); Median (Q1, Q3).

^2^ Welch Two Sample t-test; Welch Two Sample t-test; Pearson’s Chi-squared test; NA; Wilcoxon rank sum test

Bold values indicate statistical significance (p < 0.05).

### 3.1 Characteristics of the study population stratified by diminished ovarian reserve

At enrolment, 36 patients (45%) already had AMH levels compatible with diminished ovarian reserve. Compared to patients without, those with diminished ovarian reserve were slightly older (37 ± 7 vs. 34 ± 7 years, p = 0.041), had a longer disease duration (14 ± 9 vs. 10 ± 6 years, p = 0.026) and a significantly higher SLICC damage index (p = 0.007). No significant differences were observed in ethnicity, BMI, or the presence of any comorbidity, although endocrine-metabolic disorders were more frequent in patients without diminished ovarian reserve (39% vs. 22%, p = 0.12), and gynecological diseases tended to be more common in the diminished ovarian reserve group (25% vs. 9%, p = 0.055). The SLICC Damage Index was significantly higher in patients with diminished ovarian reserve than in those without, whereas SLEDAI and PGA scores were comparable between groups. Autoantibody profiles and disease activity indices were largely comparable between groups. Notably, lupus anticoagulant (LAC) positivity was more frequent among patients with diminished ovarian reserve (39% vs. 14%, p = 0.009). No other significant differences were observed for ANA, anti-dsDNA, or complement levels. HCQ use was significantly more frequent in patients without diminished ovarian reserve (89% vs 69%, p = 0.033). A higher proportion of patients with diminished ovarian reserve had prior exposure to cyclophosphamide (25% vs 9.1%, p = 0.055), although this did not reach statistical significance (**Table 2**). At study entry, 33 patients (41%) had active disease, defined as a SLEDAI-2K score ≥6, and 40 patients (50%) had active disease according to a PGA ≥1. Of these, 22 started belimumab to control disease activity.

**Table 2:** Clinical, serological, and treatment characteristics of the study population stratified by diminished ovarian reserve.

	Overall N = 80*^1^*	Diminished ovarian reserve			p-value*^1^*
Disease and laboratory features		No N = 44	Yes N = 36	Effect size (95% CI)	
ANA, n (%)	80 (100%)	44 (100%)	36 (100%)	-	
Discoid rash, n (%)	3 (3.8%)	1 (2.3%)	2 (5.6%)	OR= 2.5 (0.2, 29.1)	0.6
Photosensitivity, n (%)	21 (26%)	15 (34%)	6 (17%)	OR= 0.4 (0.1, 1.1)	0.12
Mucosal ulcers, n (%)	14 (18%)	9 (20%)	5 (14%)	OR= 0.6 (0.2, 2.1)	0.6
Synovitis, n (%)	53 (66%)	28 (64%)	25 (69%)	OR= 1.3 (0.5, 3.3)	0.6
Neurologic disorder, n (%)	4 (5.0%)	3 (6.8%)	1 (2.8%)	OR= 0.4 (0, 3.9)	0.6
Renal disorder, n (%)	27 (34%)	15 (34%)	12 (33%)	OR= 1 (0.4, 2.5)	>0.9
Serositis, n (%)	19 (24%)	9 (20%)	10 (28%)	OR= 1.5 (0.5, 4.2)	0.6
Immunologic disorder, n (%)	70 (88%)	38 (86%)	32 (89%)	OR= 1.3 (0.3, 4.9)	>0.9
Hematologic disorder, n (%)	41 (51%)	22 (50%)	19 (53%)	OR= 1.1 (0.5, 2.7)	0.8
Malar rash, n (%)	43 (54%)	21 (48%)	22 (61%)	OR= 1.7 (0.7, 4.2)	0.3
Anti-dsDNA, n (%)	68 (85%)	36 (82%)	32 (89%)	OR= 1.8 (0.5, 6.5)	0.4
C3 (mg/dl), Median (Q1, Q3)	82 (68, 100)	89 (75, 101)	80 (65, 97)	r_rb= 0.1 (-0.2, 0.3)	0.5
C4 (mg/dl), Median (Q1, Q3)	13 (8, 19)	14 (10, 21)	12 (7, 18)	r_rb= 0.1 (-0.1, 0.4)	0.3
Anti-APL, n (%)	24 (30%)	11 (25%)	13 (36%)	OR= 1.7 (0.6, 4.4)	0.3
Anti-CL IgM, n (%)	12 (15%)	8 (18%)	4 (12%)	OR= 0.6 (0.2, 2.2)	0.4
*Unknown*	2	0	2		
Anti-CL IgG, n (%)	10 (13%)	4 (9.1%)	6 (18%)	OR= 2.1 (0.6, 8.3)	0.3
*Unknown*	2	0	2		
LAC, n (%)	20 (25%)	6 (14%)	14 (39%)	OR= 4 (1.4, 12)	**0.009**
Anti-β2GPI IgM, n (%)	4 (5.2%)	3 (6.8%)	1 (3.0%)	OR= 0.4 (0, 4.3)	0.6
*Unknown*	3	0	3		
Anti-β2GPI IgG, n (%)	7 (9.1%)	3 (6.8%)	4 (12%)	OR= 1.9 (0.4, 9.1)	0.5
*Unknown*	3	0	3		
SLEDAI, Median (Q1, Q3)	4 (1, 8)	2 (0, 8)	4 (2, 6)	r_rb= 0 (-0.3, 0.2)	>0.9
PGA, Median (Q1, Q3)	0.75 (0.00, 1.50)	0.50 (0.00, 2.00)	1.00 (0.00, 1.50)	r_rb= 0 (-0.1, 0.3)	0.7
C3 reduced, n (%)	35 (44%)	17 (39%)	18 (50%)	OR= 1.6 (0.7, 3.9)	0.3
C4 reduced, n (%)	25 (32%)	11 (25%)	14 (40%)	OR= 2 (0.8, 5.2)	0.2
Active disease (SLEDAI≥6), n (%)	33 (41%)	20 (45%)	13 (36%)	OR= 0.7 (0.3, 1.7)	0.4
Active disease (PGA≥1), n (%)	40 (50%)	21 (48%)	19 (53%)	OR= 1.2 (0.5, 3)	0.7
SLICC damage index, Median (Q1, Q3)	0 (0, 1)	0 (0, 0)	0 (0, 1)	r_rb= -0.3 (-0.5, 0)	**0.007**
Treatment					
Steroid ongoing, n (%)	66 (83%)	35 (80%)	31 (86%)	OR= 1.6 (0.5, 5.3)	0.4
Hydroxychloroquine ongoing, n (%)	64 (80%)	39 (89%)	25 (69%)	OR= 0.3 (0.1, 0.9)	**0.033**
Immunosuppressive therapy ongoing, n (%)	40 (50%)	19 (43%)	21 (58%)	OR= 1.8 (0.8, 4.5)	0.2
Cyclophosphamide past exposure, n (%)	13 (16%)	4 (9.1%)	9 (25%)	OR= 3.3(0.9, 11.9)	0.055
Cyclophosphamide dose (g), Mean (SD)	8.4 (6.0)	9.4 (4.3)	7.8 (7.3)	r_rb= 0.4 (-0.3, 0.8)	0.3
Belimumab ongoing, n (%)	4 (5.0%)	2 (4.5%)	2 (5.6%)	OR= 1.2 (0.2, 9.2)	>0.9
Other biologic ongoing, n (%)	-				
Contraceptive ongoing, n/N (%)	8/80 (10%)	6/44 (14%)	2/36 (5.6%)	OR= 0.4 (0.1, 2)	0.3
Acetylsalicylic acid ongoing, n (%)	29 (36%)	16 (36%)	13 (36%)	OR= 1 (0.4, 2.5)	>0.9
Anticoagulant ongoing, n (%)	7 (8.8%)	2 (4.5%)	5 (14%)	OR= 3.4 (0.6, 18.6)	0.2
Vitamin D ongoing, n (%)	72 (90%)	41 (93%)	31 (86%)	OR= 0.5 (0.1, 2)	0.5

^1^ Wilcoxn rank sum exact test; Fisher’s exact test; Pearson’s Chi-squared test; Wilcoxon rank sum test.

Bold values indicate statistical significance (p < 0.05).

Serological profiles for herpesviruses were largely comparable between patients with and without diminished ovarian reserve (**Supplementary Table 1**). No significant differences were observed for CMV, HHV-6, HHV-7, or HHV-8 serological markers. A significantly higher prevalence of EBV IgG positivity was observed in patients without diminished ovarian reserve compared to those with diminished ovarian reserve (100% vs 86%, p = 0.016).

### 3.2 Longitudinal trends and predictors of AMH in patients with SLE

Across the 24-month follow-up (mean follow-up 22 ± 4 months), AMH levels remained overall stable, with no evidence of a progressive decline as well as the proportion of patients meeting criteria for diminished ovarian reserve based on AMH definition. Disease activity, as measured by SLEDAI and PGA, showed a progressive decrease, indicating overall clinical improvement during follow-up (**Supplementary Table 2**). Serological profiles for herpesviruses were largely stable, with persistently high IgG seroprevalence for EBV, CMV, and HHV-6. A decline in IgM positivity over time was observed across viruses, consistent with reduced rates of serological reactivation during follow-up (**Supplementary Figure 1**).

In a longitudinal analysis, AMH levels were evaluated in relation to belimumab compared with standard therapy (**Figure 1A**). The mean exposure to belimumab was 21 (± 4) months, with a mean follow-up duration of 23 (± 4) months. Despite higher baseline disease activity, patients receiving belimumab did not show decline in AMH, and trajectories remained broadly stable and comparable between groups, indicating that belimumab does not adversely affect ovarian reserve in SLE (**Figure 1B**; **Supplementary Table 3**). Given the higher baseline use of HCQ in patients without diminished ovarian reserve, a longitudinal analysis was performed to assess its effect on AMH over time. No significant time-by-treatment interaction was observed, indicating that AMH levels did not vary differently over time according to HCQ exposure (**Figure 1B**).

**Figure 1:**
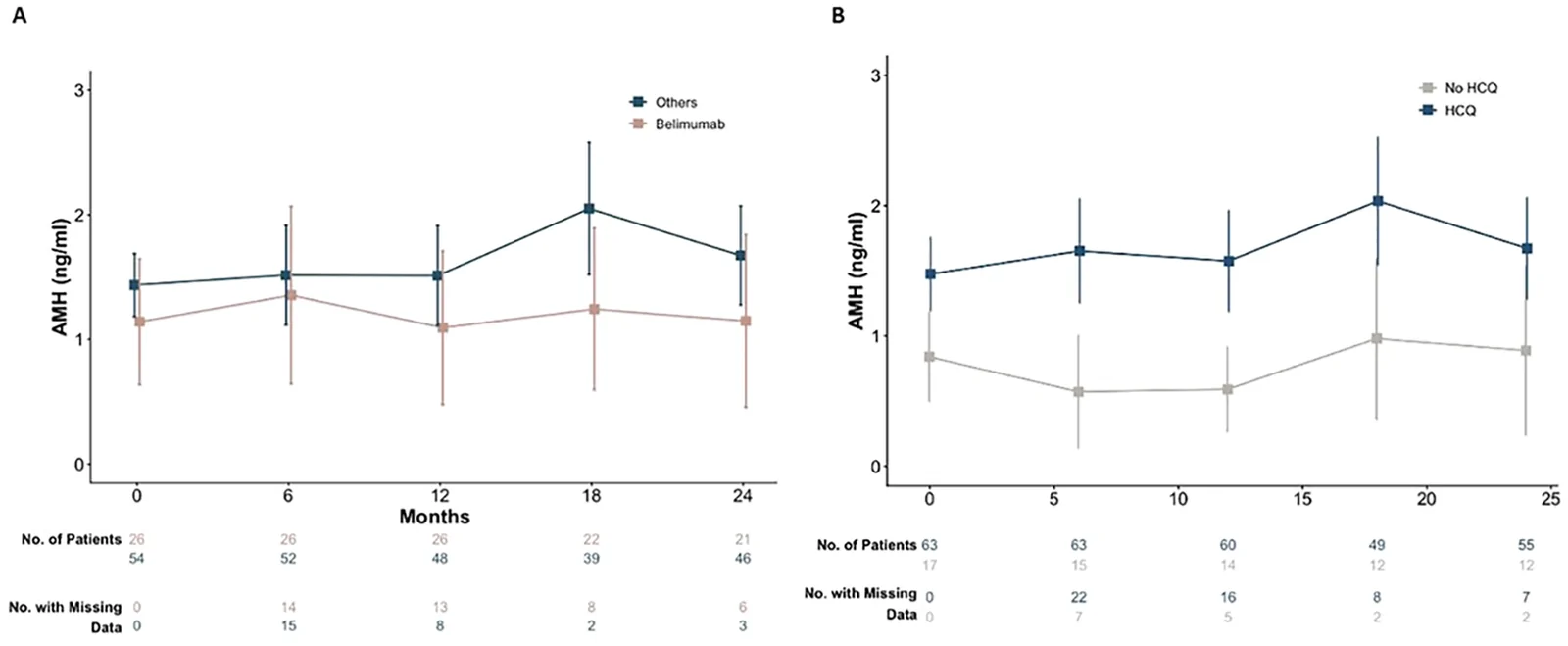
**(A)** Longitudinal changes in AMH levels in SLE patients stratified by belimumab therapy. **(B)** Longitudinal changes in AMH levels in SLE patients stratified by hydroxychloroquine therapy.

A longitudinal mixed-effects model including age at baseline, disease duration, time-varying disease activity (SLEDAI-2K), and significant baseline variables (SLICC damage index and LAC positivity) was used to evaluate AMH trajectories over time (**Table 3**). AMH levels were lower in LAC-positive patients (β = -0.582; 95% CI -1.159 to -0.006; p = 0.048) and exhibited a significant time × age interaction (β = -0.002; 95% CI -0.004 to -0.001; p = 0.008), whereas SLEDAI-2K, disease duration, and SLICC damage index were not significantly associated with AMH (all p > 0.1). In two additional models including belimumab and HCQ, no significant time × treatment interaction was observed, while age and LAC positivity remained significant predictors of AMH levels (**Table 3**).

**Table 3:** Longitudinal mixed-effects models assessing the impact of clinical characteristics, belimumab, and hydroxychloroquine on AMH levels.

*Model 1*			
Variables	Beta	95% CI	p-value
**Time (months)**	0.081	0.023, 0.140	**0.006**
**Age at baseline (years)**	-0.044	-0.083, -0.005	**0.028**
SLEDAI-2K (time-varying)	-0.024	-0.066, 0.018	0.268
Disease duration at baseline (years)	-0.022	-0.054, 0.011	0.188
SLICC damage index at baseline	-0.047	-0.311, 0.218	0.726
**LAC positivity at baseline**	-0.582	-1.159, -0.006	**0.048**
Time (months) * Age at baseline (years)	-0.002	-0.004, -0.001	**0.008**

*Model 2*			
Variables	Beta	95% CI	p-value
Time (months)	0.081	0.024, 0.138	0.005
Belimumab treatment	-0.089	-0.541, 0.362	0.698
Age at baseline (years)	-0.056	-0.092, -0.021	**0.002**
LAC positivity at baseline	-0.620	-1.154, -0.087	**0.023**
Time (months) * Belimumab treatment	-0.002	-0.028, 0.023	0.858
Time (months) * Age at baseline (years)	-0.002	-0.004, 0.000	**0.012**

*Model 3*			
Variables	Beta	95% CI	p-value
Time (months)	0.104	0.035, 0.173	0.003
Hydroxychloroquine treatment	0.462	-0.124, 1.049	0.122
Age at baseline (years)	-0.051	-0.087, -0.015	**0.006**
LAC positivity at baseline	-0.623	-1.154, -0.091	**0.022**
Time (months) * hydroxychloroquine treatment	-0.018	-0.050, 0.014	0.273
Time (months) * Age at baseline (years)	-0.002	-0.004, -0.001	**0.005**

Model 1: CI, Confidence Interval Marginal R² = 0.291; Conditional R² = 0.641.

Model 2: CI, Confidence Interval Marginal R² = 0.284; Conditional R² = 0.634.

Model 3: CI, Confidence Interval Marginal R², 0.289; Conditional R², 0.636.

Bold values indicate statistical significance (p < 0.05).

In longitudinal mixed-effects models, no significant associations were observed between AMH levels and HHV-6, HHV-7, HHV-8, or EBV serological status at baseline, nor were any significant time–serostatus interactions detected, indicating no effect on AMH trajectories (**Figure 2**). In contrast, while CMV serostatus was not associated with baseline AMH, a significant interaction with time was observed for the IgG+/IgM− profile (β = 0.038, 95% CI 0.009–0.067; p = 0.011), suggesting a modestly steeper increase in AMH over time in this subgroup (**Figure 2**).

**Figure 2:**
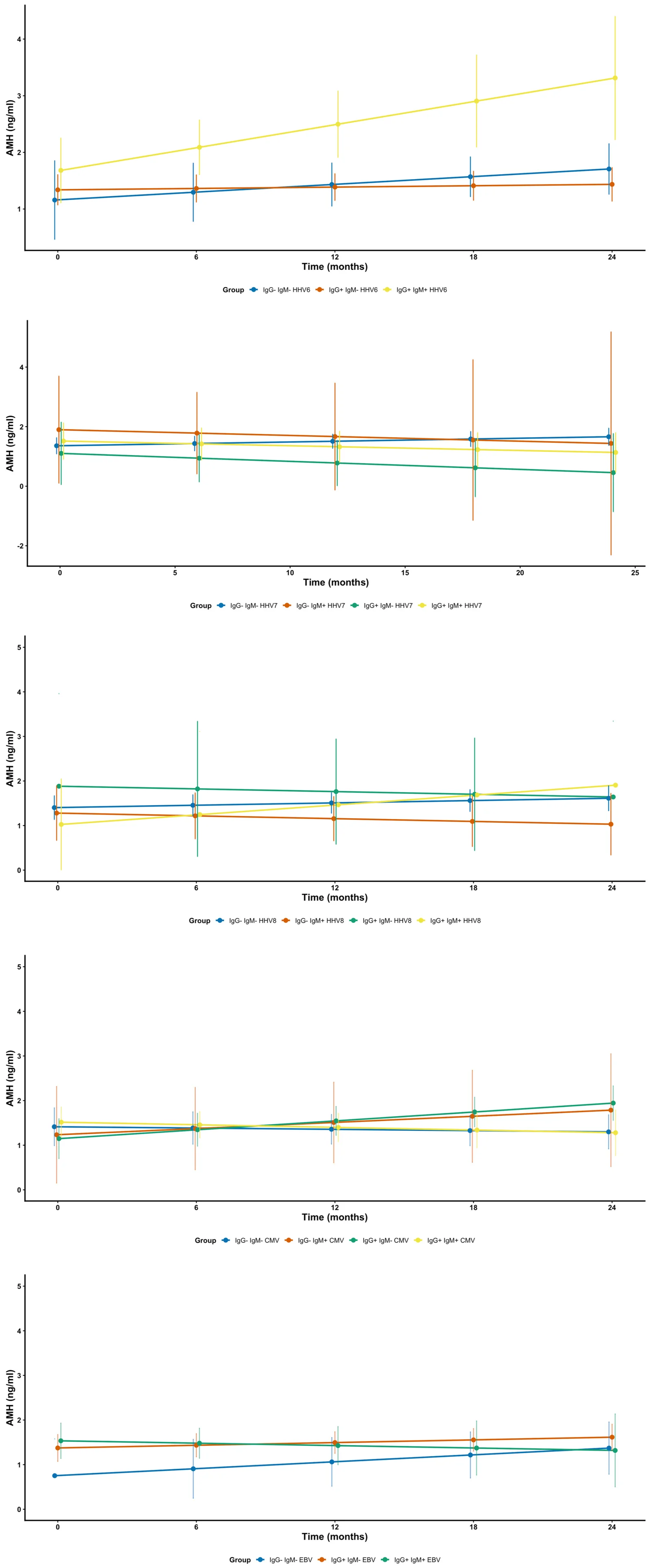
Longitudinal AMH trajectories by herpesvirus serological status.

## 4 Discussion

In this 24-months prospective longitudinal study of women of reproductive age with SLE, nearly half of the patients had diminished ovarian reserve based on AMH levels, underscoring the relevance of ovarian reserve assessment in this population. This prevalence is higher than the 5.4–17% typically reported in the literature (22), likely reflecting differences in study populations and definitions of ovarian dysfunction. Previous studies have generally relied on clinical and hormonal parameters, including menstrual irregularities, estradiol levels, and FSH, whereas our assessment was primarily based on AMH, a relatively stable marker throughout the menstrual cycle (19, 21, 23). Although elevated FSH remains the diagnostic gold standard for POI (3), it often reflects a late stage of ovarian dysfunction. Consequently, our findings suggest that low AMH levels may identify a broader spectrum of ovarian impairment that traditional markers might miss, providing a more sensitive, indication of diminished ovarian reserve in SLE patients (4, 24).

Our study identified a distinct clinical profile for patients with diminished ovarian reserve, characterized by older age, longer disease duration and higher cumulative damage as assessed by the SLICC/ACR Damage Index. The association with age reflects the physiological, non-linear decline of the follicular pool, which typically accelerates after 35 years of age (25, 26). The literature on SLE and ovarian reserve remains heterogeneous; however, our findings align with studies reporting significantly lower AMH levels in SLE patients compared with age-matched controls, even in the absence of gonadotoxic therapies, as well as reduced ovarian volume and lower antral follicle counts (27, 28). Moreover, several studies have shown that patients with reduced ovarian reserve tend to exhibit greater cumulative damage and a higher frequency of flares, further supporting the notion that chronic disease burden may contribute to ovarian impairment (29–31). Although data on cumulative damage remain limited, a recent study in adolescent females with systemic autoimmune rheumatic diseases, including SLE, identified the SLICC/ACR Damage Index as a key determinant of menstrual abnormalities (32). Conversely, other pivotal studies challenge the existence of a definitive link between SLE and diminished ovarian reserve (13, 33). Notably, LAC positivity was also associated with reduced ovarian reserve in our cohort, raising the possibility of a link between antiphospholipid antibodies and ovarian function in women with SLE. This finding is consistent with broader evidence concerning aPL antibodies and diminished ovarian reserve, as several studies have reported a strong association between aPL positivity and reduced AMH levels in both primary and secondary antiphospholipid syndrome (34–37). Although the pathogenic mechanisms through which aPLs affect ovarian function are not yet fully understood, several hypotheses have been proposed. Chen et al. suggested that these antibodies may preferentially target pre-ovulatory follicular cells, potentially exerting a direct effect on growing follicles and contributing to reduced circulating AMH levels (38). Another proposed mechanism involves aPL-mediated ovarian microvascular damage, the prothrombotic tendency associated with these antibodies may impair perfusion of small ovarian vessels, leading to subclinical ischemia and decreased AMH production (39). Overall, these findings suggest that aPL antibodies may play a pathogenic role in ovarian dysfunction through both immunological and vascular mechanisms, highlighting the need for targeted monitoring of ovarian reserve in aPL-positive women with SLE.

Despite the known impact of cytotoxic drug exposure, the safety of drug therapies represents an unresolved issue, especially concerning the newer medications currently in use. The literature identifies CYC and its cumulative dose as the primary risk factor for the development of POI (10), even if modern low-dose regimens (such as the Euro-Lupus protocol) appear to be less gonadotoxic than historical high-dose schemes (40, 41). In our cohort, prior exposure to CYC was not significantly associated with diminished ovarian reserve. This likely reflects a relatively low cumulative exposure (mean 9 ± 6.0 g), which remains below the 10 g threshold typically linked to increased ovarian damage (10, 29, 42). In contrast, a particularly noteworthy aspect of our findings concerns the role of HCQ in modulating ovarian reserve. We observed a significant association between HCQ use and preserved AMH levels. This observation is consistent with the well-established safety profile of HCQ: multiple international recommendations list it among the synthetic DMARDs considered safe for conception, pregnancy, and lactation, and its continued use is widely endorsed for women with SLE due to its association with improved maternal and fetal outcomes (43–45). Although the direct impact of HCQ on human fertility and AMH dynamics remains insufficiently characterized, evidence from animal models provides a biologically plausible framework for our results. In animal models HCQ mitigated ovarian injury and restored estrous cycles and hormone levels in chemically induced POI (46). These benefits appear to be mediated through broad immunomodulatory mechanisms, including the rebalancing of the Treg/Th17 axis and attenuation of inflammatory damage to ovarian follicles. However, data specifically addressing the direct effects of HCQ on ovarian reserve and long-term reproductive function in humans are currently lacking. In this context, the higher prevalence of HCQ use observed in patients without diminished ovarian reserve in our cohort should be interpreted with caution due to the observational nature of the study, the limited number of patients not receiving HCQ, and the possibility of residual confounding. Moreover, our longitudinal analysis did not demonstrate a significant difference in AMH trajectories according to HCQ exposure. Thus, this association should be considered hypothesis-generating and requires confirmation in larger, adequately powered longitudinal studies.

Lastly, EBV IgG seroprevalence was significantly higher in women without diminished ovarian reserve than in those with diminished ovarian reserve (100% vs. 86%), while no differences were observed for other herpesviruses. However, in the context of universally high EBV seroprevalence, this isolated association is unlikely to indicate a true biological effect and may reflect random variation.

The present study offers a novel perspective on ovarian reserve in SLE and represents, to the best of our knowledge, the first analysis to evaluate longitudinal AMH trends in patients with SLE over a 24-month period. Our findings indicate that AMH levels remain substantially stable over time, showing no measurable influence from therapies or viral serologies. In particular, the longitudinal analysis further suggests that belimumab does not exert a significant detrimental effect on ovarian reserve in this cohort, as AMH trajectories did not differ meaningfully between treated and untreated patients. This study represents one of the first attempts to investigate the impact of belimumab therapy on fertility in patients with SLE, providing novel insights into its safety profile during childbearing age. To date, available studies and case reports primarily focus on pregnancy outcomes and neonatal health, while aspects such as infertility or subfertility potentially associated with belimumab exposure remain largely unexplored. The latest 2024 recommendations from the EULAR state that belimumab should generally be discontinued before conception due to insufficient safety data, but may be considered in selected cases where disease control cannot be achieved with other compatible agents (43). Although our findings are exploratory, mixed-effects modelling suggested that biological age and lupus anticoagulant positivity were the main factors associated with AMH levels, consistent with the significant time-by-age interaction and the baseline findings, respectively. Taken together, our findings indicate that HCQ and belimumab may be compatible with the preservation of ovarian function in SLE, particularly when introduced early in the disease course. Importantly, belimumab showed no detrimental effect on AMH trajectories, which remained stable over time, while its ability to reduce cumulative organ damage may further support a favorable reproductive milieu (47, 48). These results warrant confirmation in larger, multicenter prospective cohort studies, but highlight the potential relevance of treatment strategy not only for disease control, but also for long-term reproductive outcomes, with important implications for early reproductive counselling in women with SLE.

Regarding the virological profile, the overall stability of AMH levels across EBV and HHV serostatus suggests the absence of a direct long-term impact of these pathogens on ovarian reserve. Although a statistically significant interaction emerged between CMV IgG+/IgM− serostatus and a slight increase in AMH levels over time, this observation should be interpreted with caution given the small size of this subgroup and the lack of a well-established biological rationale linking latent CMV infection to an increase in follicular reserve. Current evidence primarily links CMV infection to adverse pregnancy outcomes rather than to impaired ovarian reserve, and a direct role in female fertility remains unproven.

Despite its contributions, it is important to acknowledge that this study has some limitations. First, the assessment of diminished ovarian reserve relied primarily on AMH levels, without incorporating antral follicle counts or a comprehensive hormonal profile (e.g., FSH, LH). Consequently, the prevalence of ovarian dysfunction may have been incompletely characterized within this cohort. Second, as a single-center study with a limited sample size, statistical power was inherently limited, particularly for complex multivariable linear mixed-effects models and subgroup analyses. Therefore, point estimates and interactions derived from these models should be considered exploratory. Moreover, the predominantly Caucasian study population may limit the generalizability of our findings to more diverse SLE cohorts. Finally, a part of the study period coincided with the COVID-19 pandemic, which impacted outpatient care logistics and led to some missing data during the longitudinal follow-up.

In conclusion, this prospective longitudinal study underscores a substantial burden of diminished ovarian reserve in women of reproductive age with SLE, with biological age, disease duration, cumulative damage, and LAC positivity emerging as key determinants. While cytotoxic exposure remains the most established risk factor, hydroxychloroquine use was associated with a lower prevalence of reduced ovarian reserve, whereas belimumab was not associated with adverse longitudinal changes in AMH levels over 24 months. Despite the limitations of a single-center design and the assessment of ovarian reserve based primarily on AMH, this study provides novel insights into reproductive health in women with SLE and supports the need for early, individualized monitoring, as well as for larger prospective multicenter studies to further investigate the potential impact of treatment exposure on ovarian function.

## Data Availability

The raw data supporting the conclusions of this article will be made available by the authors, without undue reservation.
