# Supplementary material for: Diminished ovarian reserve in systemic lupus erythematosus: clinical, immunological, viral, and therapeutic determinants in a 24-month prospective study
Source: Front Immunol. 2026 Oct 14;17:1918504. doi: 10.3389/fimmu.2026.1918504 (PMC13714497; doi:10.3389/fimmu.2026.1918504)
Supplement: Supplementary file 1 [file Table1.docx]

| **Supplementary table 1.** Serological infective status of the study population divided by d**iminished ovarian reserve**. | | | | |
| --- | --- | --- | --- | --- |
|  | Overall N = 80*^1^* | **Diminished ovarian reserve** | | p-value*^2^* |
|  |  | No N = 44 | Yes N = 36 |  |
| **IgG EBV, n (%)** | **75 (94%)** | **44 (100%)** | **31 (86%)** | **0.016** |
| IgM EBV, n (%) | 28 (35%) | 16 (36%) | 12 (33%) | 0.8 |
| IgM CMV, n (%) | 40 (50%) | 22 (50%) | 18 (50%) | >0.9 |
| IgG CMV, n (%) | 55 (69%) | 31 (70%) | 24 (67%) | 0.7 |
| IgM HHV6, n (%) | 12 (15%) | 8 (18%) | 4 (11%) | 0.4 |
| IgG HHV6, n (%) | 74 (93%) | 39 (89%) | 35 (97%) | 0.2 |
| IgM HHV7, n (%) | 13 (16%) | 7 (16%) | 6 (17%) | >0.9 |
| IgG HHV7, n (%) | 15 (19%) | 8 (18%) | 7 (19%) | 0.9 |
| IgM HHV8, n (%) | 14 (18%) | 7 (16%) | 7 (19%) | 0.7 |
| IgG HHV8, n (%) | 5 (6.3%) | 2 (4.5%) | 3 (8.3%) | 0.7 |
| *^1^*Mean (SD); n (%) | | | | |
| *^2^*Wilcoxon rank sum test; Wilcoxon rank sum exact test; Fisher’s exact test; Pearson’s Chi-squared test | | | | |

**Supplementary Table 2.**

| **Longitudinal trends of hormonal levels, disease activity and serological infective status** | | | | | |
| --- | --- | --- | --- | --- | --- |
|  | 0 N = 80 | 6 N = 78 | 12 N = 74 | 18 N = 61 | 24 N = 67 |
| SLEDAI, Mean (SD) | 5 (4) | 3 (3) | 3 (3) | 2 (2) | 2 (3) |
| Unknown | 0 | 0 | 0 | 1 | 1 |
| PGA, Median (Q1, Q3) | 0.75 (0.00, 1.50) | 0.50 (0.00, 1.00) | 0.50 (0.00, 1.00) | 0.50 (0.00, 1.00) | 0.00 (0.00, 0.50) |
| Unknown | 0 | 5 | 6 | 5 | 6 |
| AMH, Mean (SD) | 1.35 (1.29) | 1.48 (1.56) | 1.41 (1.46) | 1.83 (1.68) | 1.54 (1.44) |
| IgG EBV, n (%) | 75 (94%) | 72 (92%) | 67 (91%) | 52 (85%) | 55 (82%) |
| IgM EBV, n (%) | 28 (35%) | 27 (35%) | 27 (36%) | 2 (3.3%) | 1 (1.5%) |
| IgM CMV, n (%) | 40 (50%) | 40 (51%) | 39 (53%) | 9 (15%) | 10 (15%) |
| IgG CMV, n (%) | 55 (69%) | 55 (71%) | 50 (68%) | 37 (61%) | 37 (55%) |
| IgG HHV6, n (%) | 74 (93%) | 76 (97%) | 71 (96%) | 32 (53%) | 55 (83%) |
| IgM HHV6, n (%) | 12 (15%) | 6 (7.7%) | 5 (6.8%) | 2 (3.3%) | 1 (1.5%) |
| IgM HHV7, n (%) | 13 (16%) | 12 (15%) | 13 (18%) | 6 (9.8%) | 9 (13%) |
| IgG HHV7, n (%) | 15 (19%) | 12 (15%) | 12 (16%) | 8 (13%) | 10 (15%) |
| IgM HHV8, n (%) | 14 (18%) | 11 (14%) | 11 (15%) | 5 (8.2%) | 7 (10%) |
| IgG HHV8, n (%) | 5 (6.3%) | 4 (5.1%) | 3 (4.1%) | 0 (0%) | 2 (3.0%) |
| HHV6 profile, n (%) |  |  |  |  |  |
| IgG- IgM- HHV6 | 6 (7.5%) | 2 (2.6%) | 3 (4.1%) | 28 (47%) | 11 (17%) |
| IgG+ IgM- HHV6 | 62 (78%) | 70 (90%) | 66 (89%) | 30 (50%) | 54 (82%) |
| IgG+ IgM+ HHV6 | 12 (15%) | 6 (7.7%) | 5 (6.8%) | 2 (3.3%) | 1 (1.5%) |
| Unknown | 0 | 0 | 0 | 1 | 1 |
| HHV7 profile, n (%) |  |  |  |  |  |
| IgG- IgM- HHV7 | 64 (80%) | 65 (83%) | 61 (82%) | 53 (87%) | 57 (85%) |
| IgG- IgM+ HHV7 | 1 (1.3%) | 1 (1.3%) | 1 (1.4%) | 0 (0%) | 0 (0%) |
| IgG+ IgM- HHV7 | 3 (3.8%) | 1 (1.3%) | 0 (0%) | 2 (3.3%) | 1 (1.5%) |
| IgG+ IgM+ HHV7 | 12 (15%) | 11 (14%) | 12 (16%) | 6 (9.8%) | 9 (13%) |
| HHV8 profile, n (%) |  |  |  |  |  |
| IgG- IgM- HHV8 | 65 (81%) | 66 (85%) | 61 (84%) | 56 (92%) | 58 (87%) |
| IgG- IgM+ HHV8 | 10 (13%) | 8 (10%) | 9 (12%) | 5 (8.2%) | 7 (10%) |
| IgG+ IgM- HHV8 | 1 (1.3%) | 1 (1.3%) | 1 (1.4%) | 0 (0%) | 2 (3.0%) |
| IgG+ IgM+ HHV8 | 4 (5.0%) | 3 (3.8%) | 2 (2.7%) | 0 (0%) | 0 (0%) |
| Unknown | 0 | 0 | 1 | 0 | 0 |
| **Diminished ovarian reserve** (AMH<1), n (%) | 36 (45%) | 21 (43%) | 26 (49%) | 17 (33%) | 24 (41%) |
| Unknown | 0 | 29 | 21 | 10 | 9 |

**Supplementary table 3.**

| **Baseline characteristics of the study population divided by belimumab ongoing/start therapy** | | | | |
| --- | --- | --- | --- | --- |
|  | | Belimumab ongoing/start | |  |
| Baseline characteristics | Overall N = 80^1^ | No N = 54 | Yes N = 26 | p-value^2^ |
| Age, Mean (SD) | 35 (7) | 34 (7) | 37 (8) | 0.2 |
| Ethnicity, n (%) |  |  |  | 0.4 |
| Arabic | 2 (2.5%) | 2 (3.7%) | 0 (0%) |  |
| Black/African-American | 2 (2.5%) | 1 (1.9%) | 1 (3.8%) |  |
| Caucasian | 75 (94%) | 51 (94%) | 24 (92%) |  |
| Latin-American | 1 (1.3%) | 0 (0%) | 1 (3.8%) |  |
| BMI, Mean (SD) | 23.8 (5.5) | 23.9 (4.7) | 23.6 (6.9) | 0.2 |
| Disease duration (years), Mean (SD) | 12 (8) | 11 (7) | 13 (8) | 0.4 |
| SLEDAI, Mean (SD) | 5 (4) | 3 (4) | 8 (4) | <0.001 |
| PGA, Mean (SD) | 0.96 (0.89) | 0.59 (0.71) | 1.71 (0.72) | <0.001 |
| SLICC damage index, n (%) |  |  |  | 0.042 |
| 0 | 55 (69%) | 42 (78%) | 13 (50%) |  |
| 1 | 14 (18%) | 8 (15%) | 6 (23%) |  |
| 2 | 7 (8.8%) | 2 (3.7%) | 5 (19%) |  |
| 3 | 2 (2.5%) | 1 (1.9%) | 1 (3.8%) |  |
| 4 | 2 (2.5%) | 1 (1.9%) | 1 (3.8%) |  |
| Anti-dsDNA, n (%) | 68 (85%) | 43 (80%) | 25 (96%) | 0.091 |
| C3 reduced, n (%) | 35 (44%) | 19 (35%) | 16 (62%) | 0.026 |
| C4 reduced, n (%) | 25 (32%) | 10 (19%) | 15 (60%) | <0.001 |
| APL antibodies, n (%) | 24 (30%) | 16 (30%) | 8 (31%) | >0.9 |
| Active disease (SLEDAI≥6), n (%) | 33 (41%) | 14 (26%) | 19 (73%) | <0.001 |
| Active disease (PGA≥1), n (%) | 40 (50%) | 16 (30%) | 24 (92%) | <0.001 |
| Immunosuppressive therapy ongoing, n (%) | 40 (50%) | 26 (48%) | 14 (54%) | 0.6 |
| Mycophenolate ongoing, n (%) | 18 (23%) | 12 (22%) | 6 (23%) | >0.9 |
| Azathioprine ongoing, n (%) | 16 (20%) | 10 (19%) | 6 (23%) | 0.6 |
| Methotrexate ongoing, n (%) | 2 (2.5%) | 0 (0%) | 2 (7.7%) | 0.10 |
| Cyclosporine A ongoing, n (%) | 1 (1.3%) | 1 (1.9%) | 0 (0%) | >0.9 |
| Cyclophosphamide exposure, n (%) | 13 (16%) | 8 (15%) | 5 (19%) | 0.7 |
| Cyclophosphamide dose (g), Mean (SD) | 8.4 (6.0) | 9.3 (6.5) | 6.4 (5.3) | 0.6 |
| Other immunosuppressive ongoing, n (%) | 3 (3.8%) | 3 (5.6%) | 0 (0%) | 0.5 |
| Belimumab ongoing, n (%) | 4 (5.0%) | 0 (0%) | 4 (15%) | 0.009 |
| Steroid ongoing, n (%) | 66 (83%) | 40 (74%) | 26 (100%) | 0.003 |
| Hydroxychloroquine ongoing, n (%) | 64 (80%) | 46 (85%) | 18 (69%) | 0.095 |
| Acetylsalicylic acid ongoing, n (%) | 29 (36%) | 20 (37%) | 9 (35%) | 0.8 |
| Anticoagulant ongoing, n (%) | 7 (8.8%) | 3 (5.6%) | 4 (15%) | 0.2 |
| **Diminished ovarian reserve** (AMH<1), n (%) | 36 (45%) | 22 (41%) | 14 (54%) | 0.3 |
| AMH, Mean (SD) | 1.34 (1.08) | 1.44 (0.95) | 1.14 (1.32) | 0.078 |
| ^1^Mean (SD); n (%) | | | | |
| ^2^Wilcoxon rank sum test; Fisher's exact test; Pearson's Chi-squared test | | | | |

Supplementary Figure 1. Longitudinal trends of serological infective status
